# Nutraceutical Potential of Leafy Vegetables Landraces at Microgreen, Baby, and Adult Stages of Development

**DOI:** 10.3390/foods12173173

**Published:** 2023-08-23

**Authors:** Cristina Mallor, Juan Ramón Bertolín, Pablo Paracuellos, Teresa Juan

**Affiliations:** 1 Centro de Investigación y Tecnología Agroalimentaria de Aragón (CITA), Avda. Montañana, 930, 50059 Zaragoza, Spain; jrbertolin@cita-aragon.es (J.R.B.); tjuan@cita-aragon.es (T.J.); 2 Instituto Agroalimentario de Aragón-IA2 (CITA-Universidad de Zaragoza), 50013 Zaragoza, Spain; 3 Basque Culinary Center (BCC), Paseo Juan Avelino Barriola, 101, 20009 Donostia, Spain

**Keywords:** chard, spinach, lettuce, borage, chicory, fatty acids, vitamin C, vitamin E, carotenoids, polyphenols, antioxidant activity

## Abstract

Nutraceutical compounds present in leafy vegetables have gained substantial attention due to the health benefits they offer beyond their nutritional value. The biosynthesis, composition, and concentration of these compounds vary widely among leafy vegetables and carry the influence of genetic, agronomic, and environmental factors. Recently, micro-vegetables are gaining importance among consumers worldwide and are used in gastronomy at different development stages. Another tendency is the utilization of local genetic resources as an integral component of agricultural biodiversity crucial for sustainable production. The present study identifies the nutraceutical potential of 10 leafy vegetables at the microgreen, baby, and adult development stages using local genetic resources from the Spanish Vegetable Genebank (CITA, Aragón). Specifically, two landraces for each of the following crops were used: chard (*Beta vulgaris*), spinach (*Spinacia oleracea*), lettuce (*Lactuca sativa*), borage (*Borago officinalis*), and chicory (*Cichorium intybus*). The results reinforce the value of traditional local genetics and demonstrate the potential of these leafy vegetables as a source of functional compounds (fatty acids, vitamin C, carotenoids, polyphenols, antioxidant activity, and tocopherols). The observed variability depending on the crop and the developmental stage recommends the necessity of having a varied diet, since each leafy vegetable product offers a unique nutritional profile.

## 1. Introduction

Nutraceuticals are biocompounds that are either present or have originated from food, and which offer health benefits beyond primary nutritional effects. These compounds have become more popular due to the wide range of biological activities that they provide. Among these we can find anti-cancerous, anti-inflammatory, antimicrobial, cardioprotective, hypoglycemic, and neuroprotective properties [1]. Phytocompounds obtained from dietary plant sources are an endless reservoir of nutraceutical compounds that are also present in leafy vegetables with considerable antioxidant potential [2,3]. In this way, a diet rich in green leafy vegetables can prevent cardiovascular disease [4], inhibits atherosclerosis [5], and potentially, prevent diabetes [6] and cognitive decline [7].

The biosynthesis, composition, and concentration of health-promoting compounds vary widely among leafy vegetables and carry the influence of genetic, agronomic, and environmental factors [8]. Additionally, harvest time is considered to be an influential factor in the nutraceutical composition of leafy vegetables. In recent years, microscale vegetables have become increasingly popular in homemade food preparations and have been the subject of progressively higher interest by the ready-to-eat market and the dietary supplements industry [9].

Food crops such as leafy vegetables are used to produce micro-vegetables, including those known as microgreens and baby greens. Microgreens are defined as tender, immature greens that require light for photosynthesis, a growing medium (soil or nutrient solution medium), and which represent a 7–28 day growth cycle [10]. Compared with their mature plants, baby greens are young plants with true tender leaves, with well-defined root and shoot systems, and which are harvested between 20–40 days after sowing [11].

The idea of microgreens originated in the late 1980s in San Francisco, California, and from that time until now its popularity has increased; nowadays, some of the world’s finest restaurants use them as novel culinary ingredients [12]. In this way, supply and demand of microscale vegetables is highly influenced by emerging gastronomic trends, and species selection relies on producer interaction with chefs and on consumer familiarization with their particular sensory attributes [13].

Leafy vegetables have a great potential to be adapted to a microscale and to improve the nutritional value in the human diet. One of the main advantages of microgreens and baby greens is the moderate cost of its cultivation as they only need mild treatments, do not require extensive tools and materials for growth [1], and are commonly used as raw leafy vegetables [11]. The health benefit attributions of microgreens mainly depends on their nutraceutical composition. Although many of the health benefits of mature plants consumed by humans are well-known, there is limited knowledge about the nutraceutical potential of their microgreen and baby counterparts. According to previous studies, microgreens can be considered nutraceuticals as they an excellent source of bioactive compounds [14,15,16]. Nevertheless, despite gaining importance among consumers worldwide, numerous crops and local or commercial varieties have yet not been studied.

Indigenous landraces, underutilized crops and wild edible plants constitute a vast repository for the selection of genetic material for microscale vegetable production [13].

Inclusive, resilient and sustainable food systems provide potential benefits that have been causing a global change in recent decades [17]. In terms of social sustainability, local food systems are not necessarily more resilient, but they can contribute to rural development and a sense of community [18]. Nowadays, the genetic biodiversity offers a great opportunity to solve problems related to food security and the increasing world population. In particular, traditional local genetic resources involving the local community should be explored [19].

Traditional crop cultivars called ‘landraces’ [20] play a key economic role in low-income countries, while in high-income nations they are considered to be niche products, being perceived by the consumers as high-quality products [21,22]. The characteristics of these landraces are responsible for this increasing interest; on the one hand they play an important role in the cultural heritage of local populations, and on the other hand they present some advantages such as their stability, capacity to adapt to local conditions and their nutritional composition [21,22,23]. The increasing interest in landraces has led to the definition of regional, national, or international programs dedicated to their valorization by using the important genetic diversity stored ex situ in genebanks.

The present study aims to identify the nutraceutical potential of leafy vegetables at three different stages of development (microgreen, baby green, and adult green or mature plants) using local genetic resources from the Spanish Vegetable Genebank (Centro de Investigación y Tecnología Agroalimentaria de Aragón, CITA) of five crops: chard (*Beta vulgaris*), spinach (*Spinacia oleracea*), lettuce (*Lactuca sativa*), borage (*Borago officinalis*), and chicory (*Cichorium intybus*), and two landraces for each crop. Thus, major nutritionally important phytoconstituents, including fatty acids, vitamin C, carotenoids, polyphenols, antioxidant activity, and tocopherols have been quantified.

The results demonstrate the potential of these leafy vegetables as a source of functional compounds and their variability depending on the crop and the developmental stage, reinforcing the requirement of having a varied diet, since each leafy vegetable crop stage offers a unique nutritional profile.

## 2. Materials and Methods

### 2.1. Plant Material

The plant material consisted of ten leafy vegetables from five crops: chard (*Beta vulgaris*), spinach (*Spinacia oleracea*), lettuce (*Lactuca sativa*), borage (*Borago officinalis*), and chicory (*Cichorium intybus*), and two landraces for each crop with differential traits (Table 1). Seeds were provided by the Spanish Vegetable Genebank of the Agrifood Research and Technology Centre of Aragón (BGHZ-CITA, Zaragoza, Spain) and correspond to landraces from the Aragon region of north-eastern Spain.

**Figure 1:**
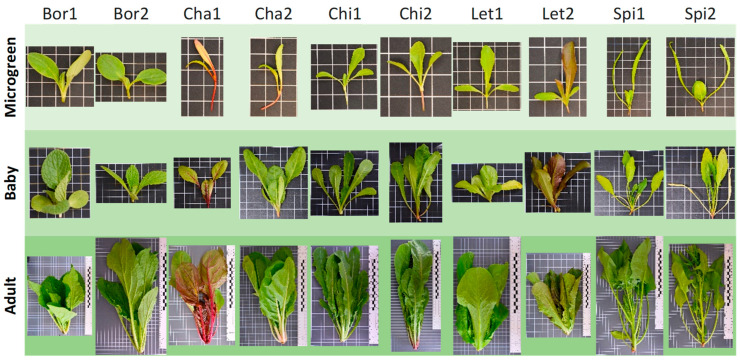
Pictures of the 10 landraces of five crops [borage (Bor), chard (Cha), chicory (Chi), lettuce (Let), and spinach (Spi)] in the 3 development stages (microgreen, baby, adult) provided by the Vegetable Germplasm Bank from CITA (Spain). The size of the grid cells in the pictures is 0.01 m × 0.01 m.

Experiments were carried out at the facilities of the Agrifood Research and Technology Centre of Aragon (CITA, Zaragoza, Spain). Seeds were sown in polystyrene seed trays filled with a mixture of peat moss and vermiculite (1:1 volume) in a greenhouse. The experiment was carried out in three replications, and the samples consisted of plants in three different development stages (microgreen, babygreen, or baby and adult or mature plants) (Figure 1). Samples were obtained by cutting plants at the substrate level, excluding roots. Microgreen samples were harvested when the plants reached the stage of expanded cotyledons and first true leaf, between 25 and 30 days after sowing. Baby samples were obtained at the two-four true leaves stage, between 50 and 60 days after sowing. After the baby samples were harvested, five seedlings per landrace were transplanted to black plastic pots 35 cm in diameter and with 26.5 L of capacity (one plant per pot), containing a substrate mixture of black and blonde peat (1:1) supplemented with NPK (20-5-10) fertilizer. Mature plants were harvested at the normal commercial stage for fresh consumption (between 85 and 95 days after transplanting).

### 2.2. Sample Preparation

A total of 90 samples were obtained from three replications, three development stages and ten varieties, as previously described. For the microgreens and baby samples, 3 different replicates (70 g of fresh weight per replica) were obtained by randomly grouping seedlings. For mature plants, intermediate leaves from three plants (one plant per replicate) were pooled. Fresh samples were weighed in microperforated bags and lyophilized. Once lyophilized, these samples were weighed, ground to 0.2 mm with an ultra-centrifugal mill (Rotary Mill, ZM200; Retsch, Haan, Germany) and stored at −80 °C protected from light until analyses.

### 2.3. Composition Analysis

#### 2.3.1. Fatty Acids

The fatty acids (FAs) profile was determined as fatty acid methyl esters (FAMEs) by using gas chromatography with a flame ionization detector, following the method described in Sukhija and Palmquist [24]. Briefly, FAs of 500 mg of freeze-dried samples were methylated with 4 mL of 0.5 M sodium methoxide in methanol and 4 mL of 1/10 *v*/*v* acetyl chloride in methanol consecutively, and extracted with 3 mL of heptane with 1 mg of nonadecanoic acid (C19:0) as internal standard. In order to remove traces of water and pigments, the extracts were purified with anhydrous sodium sulfate and activated carbon before injection into the gas chromatograph. FAMEs determination was achieved in a Bruker Scion 436-GC (Bruker, Billerica, MA, USA) gas chromatograph equipped with a CP-8400 autosampler and an SP-2560 capillary column (100 m × 0.25 mm × 0.2 μm) (Supelco, St. Louis, MO, USA). The FAMEs identification was realized by comparison with the relative chromatographic retention times of the standard FAME mixtures GLC-401, GLC-463, GLC-532, GLC-538, GLC-643, and GLC-642 (Nu-Chek Prep, Elysian, MN, USA). Quantification was performed as described in ISO 12966-4:2015 [25].

#### 2.3.2. Carotenoids and Tocopherols

Carotenoids and tocopherols were determined by liquid chromatography with a photodiode array and fluorescence detector, respectively, as described by Blanco et al. [26]. Briefly, 50 mg of the samples were extracted 3 times with 3 mL of methanol–acetone–petroleum ether (1-1-1 *v*-*v*-*v*, 0.01% butylhydroxytoluene) solution until a white pellet was obtained. After this, the supernatants were evaporated in a vacuum evaporator. The dry residues were dissolved in 3 mL of acetonitrile–dichloromethane–methanol (75–10–15), and 1 mL of each was filtered through a 0.2 μm × 13 mm of polytetrafluoroethylene (PTFE) filter and transferred into a 2 mL amber glass vial. Carotenoids and tocopherols were determined into an ACQUITY UPLC H-Class liquid chromatograph (Waters, Milford, MA, USA), coupled to an absorbance detector (Acquity UPLC Photodiode Array PDA eλ Detector; Waters, Milford, MA, USA), a fluorescence detector (2475 Multi λ Fluorescence Detector; Waters) and controlled by Empower3 software (Waters). Separation was carried out on an Acquity UPLC HSS T3 column (150 mm × 2.1 mm ×1.8 μm × 2.1 mm; Waters). Detection of carotenoids was undertaken by absorbance at a wavelength of 450 nm. Detection of tocopherol homologues was undertaken by fluorescence under an excitation wavelength of 295 nm and emission of 325 nm. β-carotene, lutein and tocopherol homologues were identified by comparison of their retention times and spectral analyses, and quantified by external calibration with those pure standards. β-carotene (97% purity), lutein (97% purity), and tocopherols (99% purity α-tocopherol, 97% purity γ-tocopherol, 97% purity δ-tocopherol) were purchased from Sigma-Aldrich (St. Louis, MO, USA) and concentrations of β-carotene, lutein, α-tocopherol, γ–tocopherol, and δ-tocopherol standard solutions were calculated before use by absorbance of each solution using molar absorption coefficients, as previously reported [27]. Zeaxanthin, neoxanthin, violaxanthin, 13 Z-β-carotene, and 9 Z-β-carotene were identified by comparison of their retention times and spectral analyses, as previously reported [28,29], and quantified (semiquantitative analysis) by calculating response factors to β–carotene calibration based on their molar absorption coefficients [28].

#### 2.3.3. Vitamin C

The vitamin C as sum of ascorbic acid (AA) and dehydroascorbic acid (DHAA) was determined by liquid chromatography with a photodiode array detector using the method described by Medina-Lozano et al. [30]. Briefly, AA and DHAA of 50 mg of freeze-dried samples were extracted with 5 mL of solvent extraction solution: 8% acetic acid (*v*/*v*), 1% MPA (meta-phosphoric acid) (*w*/*v*), and 1 mM EDTA (ethylenediaminetetraacetic acid) in ultrapure water. Then, the supernatants were filtered through a 0.2 μm × 15 mm of regenerated cellulose filter and stored in an amber glass vial. For the AA determination, 200 µL of the extracts were diluted with 800 µL of ultrapure water before injection. In the case of DHAA determination, DHAA was reduced to AA before injection, to which 200 µL of the reducing solution 40 mM DTT (1,4-Dithiothreitol) with 0.5 M Tris pH 9.0 was added to 200 µL of the extracts, leaving them to react for 30 min at room temperature and in darkness. Afterwards, 200 µL of 0.4 M H_2_SO_4_ and 400 µL of ultrapure water was added. In this case, this extract contains the sum of total vitamin C (AA + DHAA) as AA form. Vitamin C was determined into an ACQUITY UPLC H-Class liquid chromatograph (Waters, Milford, MA, USA) equipped with a silica-based bonded phase column (Acquity UPLC HSS T3, 1.8 μm × 2.1 mm × 150 mm column; Waters), an absorbance detector (Acquity UPLC Photodiode Array PDA eλ Detector; Waters), and controlled by Empower3 software (Waters). Vitamin C as AA form was detected by absorbance at λ = 245 nm and quantified using an external calibration curve of five standards ([AA] = 0.5–25 µg mL^−1^). Ascorbic acid (≥99.9% purity) was purchased from Sigma Aldrich (St. Louis, MO, USA).

#### 2.3.4. Total Polyphenols

Total polyphenols were determined spectrophotometrically by the Folin–Ciocalteu method, following the method described in Makkar [31]. Briefly, 200 mg of samples was extracted twice with 5 mL of acetone/ultrapure water/formic acid (47.5-47.5-5, *v*-*v*-*v*). Both extracts were mixed and 30 μL of these were diluted to 1 mL with ultrapure water. To the diluted extracts, 0.5 mL of the Folin–Ciocalteu reagent and 2.5 mL of 20% *w*/*v* of calcium carbonate in ultrapure water were added. The mixtures were shaken and allowed to react for 35 min at room temperature and protected from light. Finally, the absorbance of the samples was measured at 725 nm in a Heλios β spectrophotometer (Thermo Scientific, Waltham, Massachusetts, USA). The quantification was performed by external calibration curve with tannic acid standards ([Tannic acid] = 0–60 mg L^−1^).

Table S1 summarizes the plant species utilized for the present study, as well as the developmental stages analyzed and all the compounds quantified.

#### 2.3.5. Antioxidant Activity

The antioxidant activities (AO) were determined with the total radical scavenging activity [2,2′-azino-bis (3-ethylbenzothiazoline-6-sulfonic acid) (ABTS)] based on the method of Rufino-Moya et al. [32]. We worked with the same diluted extracts for the total polyphenols analysis. In this way, 20 μL of the diluted extracts and 280 μL of ABTS solution in absolute ethanol (absorbance value of 0.700 ± 0.02 at 730 nm) were mixed for 30 min at room temperature. Then, absorbance at 730 nm was measured using an EPOCH microplate spectrophotometer (BioTek, Winooski, VT, USA). The quantification was carried out by external calibration curve with Trolox standards ([Trolox] = 0–0.4 μmol mL^−1^).

### 2.4. Statistical Analysis

Data for each parameter were taken in three replicates for each of the three developmental stages and ten landraces. The significance of variables was assessed by analysis of variance (ANOVA), followed by a post-hoc Tukey B test to construct homogeneous groups. The differences between individual means were deemed to be significant at *p* < 0.05. For each parameter, the one-way ANOVA was used to determine whether there were any statistically significant differences between the means of the three developmental stages for each of the 10 studied landraces, and between the means of the 10 landraces for each of the developmental stages. All analyses were performed using the SPSS statistical package (SPSS for Windows, version 16.0).

## 3. Results and Discussion

### 3.1. Fatty Acids

The study identified 12 fatty acids: myristic acid (MA, C14:0), palmitic acid (PA, C16:0), palmitoleic acid (PLA, C16:1 9c), roughanic acid (RA, C16:3), margaric acid (C17:0), stearic acid (STA, C18:0), oleic acid (OA, C18:1 9c), cis-vaccenic acid (C18:1 11c), linoleic acid (LA, C18:2 n-6), alfa-linolenic acid (ALA, C18:3 n-3), gamma-linolenic acid (GLA, C18:3 n-6), and stearidonic acid (SDA, C18:4, n-3) (Table S2).

We can highlight that RA has only been detected in chard (1.50–2.00%) and spinach (5.12–7.33%), while SDA has only been detected in borage. RA is an omega-3 fatty acid abundant in many plant species [33]. Nevertheless, our results indicated that we were not able to detect it in borage, lettuce, and chicory; it was only detected in species belonging to the botanical family *Chenopodiaceae* (spinach and chard) according to Mongrand et al. [34].

SDA, a metabolite of ALA via D6-desaturase, has been previously found in borage [35,36,37]. Our results showed a range from 10.29% (Bor2, microgreen) to 14.16% (Bor1, baby), lower than those previously obtained by Peiretti et al. (24%) [35] or by Stahlër et al. (25%) [36], and similar to the values obtained by Montaner et al. [37], which varied from 7.3% to 14.6%.

**Figure 2:**
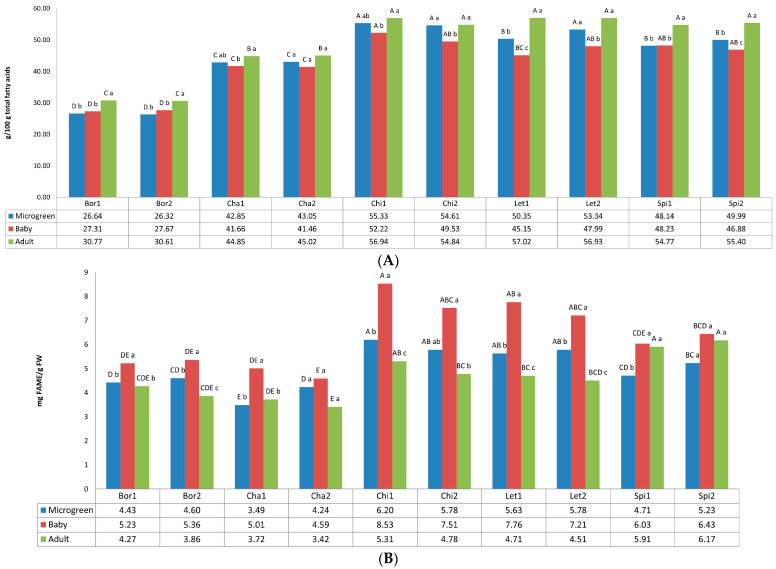
Fatty acids content in the 10 landraces of 5 leafy vegetable crops (borage “Bor”, chard “Cha”, chicory “Chi”, lettuce “Let”, and spinach “Spi”) evaluated at 3 developmental stages (microgreen, baby, and adult). (**A**) Alpha-linolenic acid (ALA, C18:3 n-3). (**B**) Fatty acid methyl esters (FAME). Values are the mean of three replicates, subjected to a one-way ANOVA. Different capital and lowercase letters indicate significant differences between landraces for each developmental stage and between developmental stages for each landrace, respectively, at *p* < 0.05 by Tukey B test. FW: Fresh weight.

Figure 2 shows the total fatty acids, expressed as fatty acid methyl esters (FAME), and the alpha-linolenic acid (ALA) content, the major fatty acid detected. Both FAME and ALA contents differed statistically according to the variety and the developmental stage of the analyzed plants.

The values for the essential fatty acid ALA varied from 26.32% (Bor2, microgreen) to 57.02% (Let1, adult). Borage presented the lowest values for ALA content in comparison with the other four crops, at all developmental stages. Among borage samples, the values differed according to the developmental stage, showing the lowest contents at the microgreen and baby stages. Montaner et al. [37] found in a borage germplasm collection a range of ALA in leaf blades between 34.70% and 41.59%, depending on the genotype and the harvest date, which are higher than our values of 30.77% and 30.61% for adult plants of Bor1 and Bor2, respectively. The difference may be because Montaner et al. [37] did not include the petioles of the leaves, which were analyzed separately, obtaining lower values for ALA (between 14.85% and 31.57%). Regarding microgreens, major contents have been found for both chicory landraces (Chi1 and Chi2) and one of the lettuce landraces (Let2); at the baby stage the highest values corresponded to chicory (Chi1 and Chi2), spinach (Spi1 and Spi2), and lettuce (Let2); finally, in adult plants, chicory (Chi1 and Chi2), spinach (Spi1 and Spi2), and lettuce (Let1 and Let2) showed the highest values. Our data for the baby stage (except borage), ranging from 41.46% to 52.22%, agree with those published previously for Korean baby-leaf vegetables, ranging from 44.73 to 54.39% [38]. ALA has been reported as the major fatty acid in wild *Cichorium intybus* with values of 60.45% [39], slightly higher than those found in our study for this species of 56.94 and 54.84% for adult plants of Chi1 and Chi2, respectively. ALA plays a vital role in the human diet, since it is the precursor for long-chain n-3 fatty acids. By itself, it may have beneficial effects for human health in the control of chronic diseases [40]. Green leafy vegetables and herbs have been established as a potential source of ALA [41]. Flaxseed, walnuts, and canola oil are considered to be a rich and natural source of ALA, which contain 28.8, 9.1 and 9.1% ALA, respectively [42]. Compared to this, in the present study the total lipids extracted from different leafy vegetables are found to contain up to 57.02% of ALA, which is much higher than known sources. Specifically, both chicory landraces have been shown to be the best source of ALA at all developmental stages.

In reference to other fatty acids, we can highlight the GLA content in borage (Table S2). While all samples showed values less than 0.2%, borage presented GLA contents of 12.26% and 11.11% for Bor1 and Bor2 microgreens, respectively. Adult borages presented lower contents of 8.31% and 8.17%, which are in the range found in borage leaf blades between 4.63% and 10.98% by Montaner et al. [37]. The presence in borage of the unusual enzyme D6-desaturase allows the synthesis of GLA and SDA [37,43]. Both fatty acids have been found in this study, differing with the pattern of the other crops. In fact, borage has been described as a good source of GLA and SDA, which are fatty acids that are not commonly found in plants [37].

FAME contents varied from 3.42 mg/g Fresh Weight (FW) (Cha2, adult) to 8.53 mg/g FW (Chi1, baby). Both landraces belonging to the same crop showed similar contents at the three stages, and all varieties showed higher values at the baby stage. In general, chicory and lettuce landraces showed the highest FAME contents, while borage and chard exhibited the lowest contents. Fatty acids are one of the structural components of cell membranes and provide one of the general defense systems against various biotic and abiotic stresses [44]. As Ćavar Zeljković et al. [45] stated for lettuce, fatty acid levels significantly vary depending on stress and its intensity. Our results also show that the development stage of the plants significantly influence the fatty acid content, being lower at the adult stage in comparison with the microgreen and baby developmental stages. Fatty acids influence health, well-being, and risk of disease. Green leafy vegetables are not a rich source of dietary fats; however, they are well known for their high content of polyunsaturated fatty acids (PUFAs), primarily in the form of ALA and LA [46]. Our results show that the baby stage is the best regarding the fatty acid content, with values that ranged from 4.59 mg/g FW (chard) to 8.53 mg/g FW (chicory).

As expected, unsaturated fatty acids (UFA) predominate over saturated fatty acids (SFA). In all samples, PUFA, with a mean value of 70.66%, predominates over MUFA due to the contribution of 19.00% LA and 45.53% ALA. Besides, we can highlight the contribution of GLA in borage (mean 12.13%) and RA in chard (mean 1.75%) and spinach (mean 6.20%) (Table S2). SFA included the main contribution of PA and STA.

Interestingly, in line with the findings of Petropoulos et al. [47] for wild chicory (*Cichorium spinosum*) and Montaner et al. [37] for borage, the detected ratios of PUFA/SFA and n-6/n-3 fatty acids at the various growth stages of the present study shows health benefits. The PUFA/SFA ratio varied from 2.12 (Bor2, baby) to 3.68 (Let2, adult), so all of the values were higher than the 0.45 suggested for good nutritional quality [46]. In reference to n6/n3, the ratio varied from 0.19 (Spi2, adult) to 0.75 (Bor2, microgreen). According to Simopoulos [48], this ratio should be lower than 4.0 to reducing the risk of many of the chronic diseases that are of high prevalence in Western societies. These results reinforce the fact that studied leafy vegetables at different development stages have a good nutritional quality.

### 3.2. Carotenoids

The study identified neoxanthin, violaxanthin, zeaxanthin, lutein, 13-Z-β-carotene, 9-Z-β-carotene, all-E-β-carotene, and β-carotene total (Table S3).

Lutein and zeaxanthin are xanthophyll carotenoids, accumulating in the macula of human eyes. Numerous epidemiological studies have shown that lutein and zeaxanthin play a critical role in the prevention of age-related macular degeneration and cataracts [49]. Given lutein’s and zeaxanthin’s antioxidant and anti-inflammatory properties, it is hypothesized that these carotenoids may also have beneficial effects on diseases for which oxidation and inflammation play a role [50]. In our study, all samples presented lutein and zeaxanthin at all developmental stages, presenting mean values of 37.38 µg/g FW and 3.25 µg/g FW, respectively. Regarding lutein content, all samples presented higher values at the microgreen stage, except one spinach landrace which showed the highest value of 70.39 µg/g FW in adult plants. Zeaxanthin contents were also higher in microgreens, except for spinach, which presented higher values in adult plants. These findings agree with Xiao et al. [14] who suggest that immature leaves of the 25 analyzed microgreens tend to possess a higher lutein and zeaxanthin concentration than their fully grown plant counterparts. It is worth noting the value obtained for microgreens of chicory that presented lutein levels of 56.83 µg/g FW and 58.97 µg/g FW for Chi1 and Chi2, respectively, and microgreens of lettuce that presented zeaxanthin contents of 7.33 µg/g FW and 6.12 µg/g FW.

Violaxanthin is a natural, orange-colored carotenoid found in the photosynthetic organs of plants [14]. Our results showed higher contents in microgreens than in their baby and adult counterparts. The violaxanthin content varied from 11.08 µg/g FW (Cha2, adult) to 50.63 µg/g FW (Chi1, microgreen). Previous research showed mean values for chicory of 22.4 ± 1.3 µg/g FW [51], which are similar to the values found in this study for adult chicories of 26.7 µg/g FW and 19.56 µg/g FW for Chi1 and Chi2, respectively. These values correspond to half the value found in the microgreens’ counterparts. Spinach is considered to be a good source of violaxanthin [52], with reported values of 29.9 µg/g FW and 37.1 µg/g FW by Lester et al. [53]. Our results showed higher values for Spi1 and Spi2, of 44.18 µg/g FW and 47.35 µg/g FW, respectively. Nevertheless, our results also showed higher levels of violaxanthin for chicory and lettuce microgreens.

**Figure 3:**
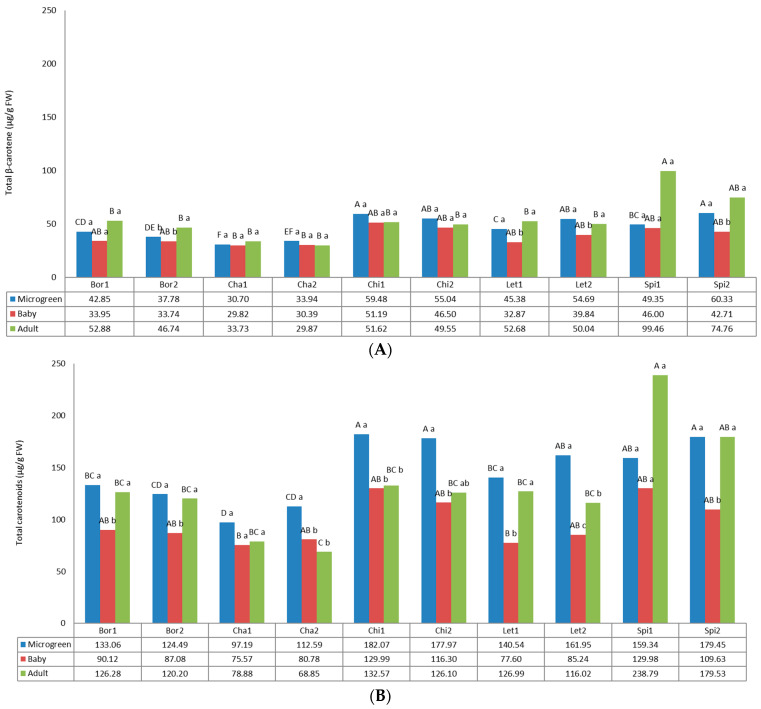
Carotenoids content in the 10 landraces of 5 leafy vegetable crops (borage “Bor”, chard “Cha”, chicory “Chi”, lettuce “Let”, and spinach “Spi”) evaluated at 3 developmental stages (microgreen, baby and adult). (**A**) Beta-carotene. (**B**) Total carotenoids. Values are the mean of three replicates, subjected to a one-way ANOVA. Different capital and lowercase letters indicate significant differences between landraces for each developmental stage and between developmental stages for each landrace, respectively, at *p* < 0.05 by Tukey B test. FW: Fresh weight.

Figure 3 shows the total β-carotene content, one of the most important carotenoids, and the total carotenoids. Both parameters statistically differed according to the variety and the developmental stage of the analyzed plants.

For β-carotene, the values varied from 29.81 µg/g FW (Cha1, baby) to 99.47 µg/g FW (Spi1, adult). Both chard landraces presented the lowest values, showing similar values at the three developmental stages. In general, chicory and spinach presented the highest values, highlighting the great content of spinach at the adult stage. β-carotene is an important fat-soluble antioxidant and can protect cellular membranes by scavenging free radicals [14]. According to the results obtained by Montefusco et al. [54], β-carotene varied in cultivated and wild chicory cultivars from 3.3 to 14.1 µg/g, much lower that values found for adult plants in this study, between 51.62 and 49.56 µg/g for Chi1 and Chi2, respectively. Our results are similar to values found by Reif et al. [55] in the cv. Charlotte of the flavescens chicory variety (44.8 µg/g FW). However, this concentration is five times higher than the one found by Mzoughi et al. [56]. Gamba et al. [57] stated that this difference could be due to the plant varieties or the type of solvents used to extract the chemicals. Regarding spinach, our values in baby plants (46.00 µg/g FW and 42.71 µg/g FW for Spi1 and Spi2, respectively) are lower those previously reported by Lester et al. [53] for two cultivars of baby leaf spinach of 55.9 µg/g FW and 61.3 µg/g FW, respectively.

For total carotenoids, the values varied from 68.85 µg/g FW (Cha2, adult) to 238.79 µg/g FW (Spi1, adult). As for β-carotene, chard exhibited the lowest values of total carotenoids, while spinach showed the highest values. In general, baby plants exhibited lower values than microgreens and adult plants, and most of the local landraces exhibited the highest values for microgreens (Bor1, Bor2, Cha2, Chi1, Chi2 and Let2). Microgreens of chicory presented the higher values of 182.07 µg/g FW and 177.97 µg/g FW for Chi1 and Chi2, respectively. Previous total- β-carotene content reported for chicory adult plants of 244.1 µg/g FW [51] was higher than those found in this study of 132.57 µg/g FW and 126.10 µg/g FW for Chi1 and Chi2, respectively. Our results disagree with those previously obtained by Martínez-Ispizua et al. [58], because they reported higher carotenoid contents for lettuce at the baby stage in comparison with the microgreen and adult stages, while our results showed that lettuce babies had the lowest content. It has been suggested that differences in the carotenoids content between lettuce types is related to the head structure because it is regulated by light [59,60]. The range obtained for carotenoids (68.85–238.79 µg/g FW) is comparable or even much higher to other crops known for their high carotenoid contents, such as carrots (95.9 µg/g FW) [61] and red peppers (63–130 µg/g FW) [62,63].

### 3.3. Tocopherols

**Figure 4:**
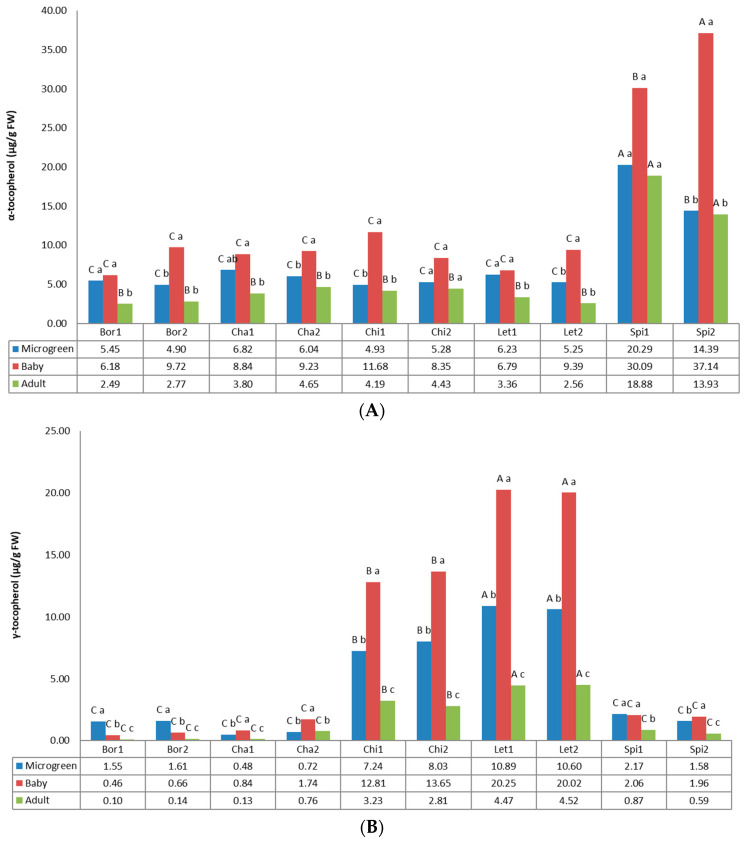

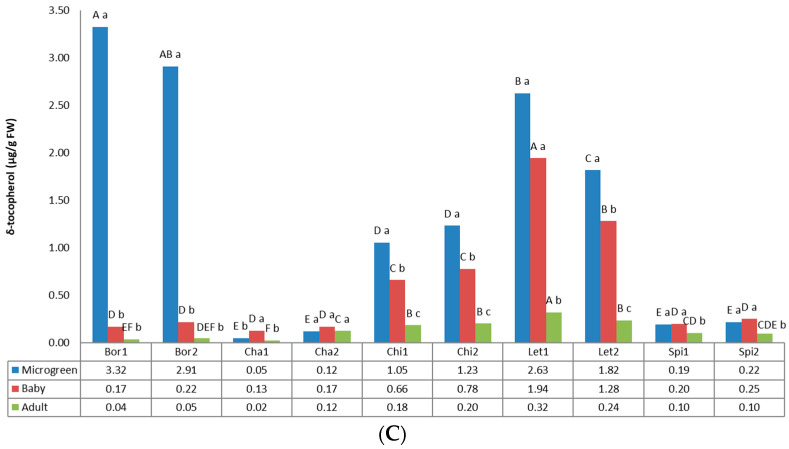
Tocopherols content in the 10 landraces of 5 leafy vegetable crops (borage “Bor”, chard “Cha”, chicory “Chi”, lettuce “Let”, and spinach “Spi”) evaluated at 3 developmental stages (microgreen, baby, and adult). (**A**) α-tocopherol. (**B**) γ-tocopherol. (**C**) δ-tocopherol. Values are the mean of three replicates, subjected to a one-way ANOVA. Different capital and lowercase letters indicate significant differences between landraces for each developmental stage and between developmental stages for each landrace, respectively, at *p* < 0.05 by Tukey B test. FW: Fresh weight.

Tocopherols and tocotrienols are together summarized as vitamin E, known as fat-soluble antioxidants, and each group is formed by four isomers (α, β, γ, and δ) [64]. Figure 4 shows tocopherol contents according to α-tocopherol, γ-tocopherol, and δ-tocopherol values. Tocopherol content was significantly affected by the growth stage and cultivar. The main detected tocopherols were α-tocopherol and γ-tocopherol, except for borage, in which microgreens showed higher levels of δ-tocopherol than γ-tocopherol.

For α-tocopherol, the values ranged from 2.49 µg/g FW (Bor1, adult) to 37.14 µg/g FW (Spi2, baby). Among the local cultivars, both spinach landraces stood out from the others due to their high content of this compound, specifically at the baby stage. In fact, baby samples also showed the highest values in the other local cultivars in comparison to the microgreen and adult stages. Previous studies have shown in spinach microgreens α-tocopherol content of 14.2 mg/100 g [14] and 17.1 mg/100 g FW [65], which are much higher than those obtained in this study for Spi2 and Spi1 of 14.39 µg/g FW and 20.29 µg/g FW. Values obtained for other species also differed from previous studies. As examples, our values for chicory (4.93–5.28 µg/g FW) and lettuce (6.23–5.25 µg/g FW) clearly differed from those obtained by Paradiso et al. [66] of 22.6 µg/g FW and 2.2 µg/g FW, respectively. Among others, the variety has been stated as a factor of variation in α-tocopherol content. Saini et al. [38] reported in a study on baby leafy vegetables a range for lettuce from 7.10 µg/g FW (Romaine green lettuce) to 28.00 µg/g FW (Batavian lettuce). The α-tocopherol is the most common and biologically active form of vitamin E, and has been reported as an important phytochemical that is present in microgreens. It forms part of the fat-soluble antioxidant system of the cell, inhibiting in vivo lipid oxidation [11,67]. In this way, the obtained results suggest that spinach is our best source of α-tocopherol, specially at the baby stage.

For γ-tocopherol, the values varied from 0.10 µg/g FW (Bor1, adult) to 20.24 µg/g FW (Let1, baby). Lettuce and chicory were the crops that exhibited higher values, clearly standing out from the others and highlighting the content at the baby stage, followed by the microgreen and adult stages. Values found for baby lettuces (20.25–20.02 µg/g FW) are similar to those previously reported for Batavian lettuce of 19.68 µg/g FW, but higher than that reported for other lettuce cultivars, such as Romaine green and Romaine red of 9.71 and 9.78 µg/g FW, respectively [38].

The most active form of all the tocopherols is α-tocopherol, while γ-tocopherol is the most abundant form in plants [68]. Xiao et al. [14] found 25 varieties of microgreens values that ranged from 4.9 to 87.4 and from 3.0 to 39.4 mg/100 g FW. In this study, α-and γ-tocopherol contents for the 10 different landraces of microgreens varied from 4.9 µg/g FW (Bor2) to 20.29 µg/g FW (Spi1) and from 0.48 µg/g FW (Cha1) to 10.89 µg/g FW (Let1).

For δ-tocopherol, the values varied from 3.32 µg/g FW (Bor2, microgreen) to 0.02 µg/g FW (Cha1, adult). The higher contents were found for microgreens of borage and lettuce crops. In borage, the content of δ-tocopherol decreased to a great extent at the next development stage, from values between 3.32 and 2.91 µg/g FW at microgreen stage to 0.17–0.22 µg/g FW at the baby stage and 0.04–0.05 µg/g FW at the adult stage. This decrease was also evident in lettuce and chicory samples, although there were not such high differences between microgreen and baby contents as in borage. Spinach and chard samples showed the lowest values for this compound.

Various studies evidence the antioxidant properties of tocopherols, suggesting their essential role in human health. They can protect against oxidative damages in many tissues and play an important role in muscle or immune functions. At the same time, tocopherol supplementation could prevent many age-related diseases such as cardiovascular or Alzheimer’s disease [69]. Various researchers reported that microgreens contain a substantial amount of vitamin E [14,66,70], higher than their respective mature leaves [65]. Our results support these findings, showing that, in general, microgreens and babies contain considerably higher concentrations of α-tocopherol, γ-tocopherol, and δ-tocopherol than their mature counterparts, highlighting their potential as a functional food.

### 3.4. Vitamin C

**Figure 5:**
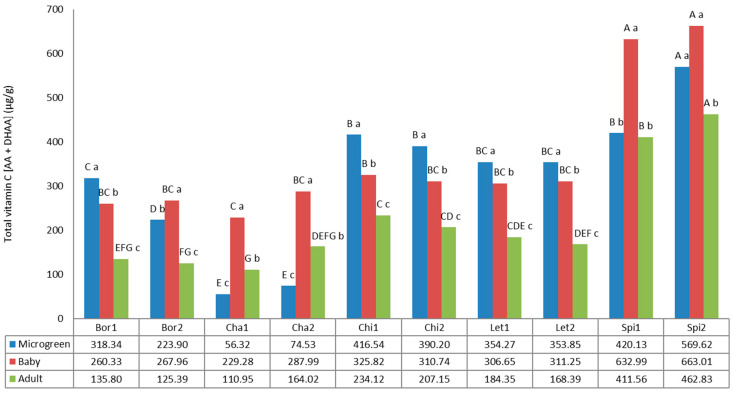
Vitamin C content in the 10 landraces of 5 leafy vegetable crops (borage “Bor”, chard “Cha”, chicory “Chi”, lettuce “Let”, and spinach “Spi”) evaluated at 3 developmental stages (microgreen, baby, and adult). Values are the mean of three replicates, subjected to a one-way ANOVA. Different capital and lowercase letters indicate significant differences between landraces for each developmental stage and between developmental stages for each landrace, respectively, at *p* < 0.05 by Tukey B test. FW: Fresh weight.

Ascorbic acid, also known as vitamin C, is an essential nutrient for humans, considered the most valuable antioxidant for living beings [71]. Ascorbic acid is also the most effective absorption enhancer of non-heme iron [65]. Figure 5 shows total vitamin C or total ascorbic acid (TAA), calculated as the summatory of free ascorbic acid (FAA) and dehydroascorbic acid (DAA). The values for vitamin C statistically differed according to the variety and the developmental stage of the analyzed plants.

The results showed a wide range of variation from 56.32 µg/g FW (Cha1, microgreen) to 663.01 µg/g FW (Spi2, baby). The highest values were found for spinach local cultivars, highlighting the values for baby plants, which were higher than microgreen and adult developmental stages. The same tendency was found for chard, which also showed the highest contents at the baby stage. In contrast, borage, chicory, and lettuce showed highest values at the microgreen stage, following by the baby and adult stages, except for Bor2. Xiao et al. [14] showed TAA concentrations ranging from 20.4 mg/100 g FW (sorrel) to 147.0 mg/100 g FW (red cabbage) for 25 microgreens, while Dhaka et al. [72] reported values for ascorbic acid of six diverse microgreens species ranging from 28.48 mg/100 g FW (red radish) to 140.22 mg/100 g FW (red cabbage). Only values obtained for chard of 56.32 and 74.53 µg/g FW, respectively, are out of these ranges. The other species showed TAA concentrations from 223.9 µg/g FW (Bor2) to 569.62 µg/g FW (Spi2). On the other hand, the obtained results for borage microgreens (318.34 and 223.90 µg/g FW, for Bor1 and Bor2, respectively) are lower than previously reported for this species of 85.27 mg/100 g FW ascorbic acid [73]; the obtained values for spinach microgreens (420.13 and 569.62 for Spi1 and Spi2, respectively) are also lower than previously reported of 71.2 mg/100 g FW ascorbic acid by Ghoora et al. [65]; and the obtained values for lettuce microgreens (354.27 and 353.85 µg/g FW, for Let1 and Let2, respectively) were in the range previously reported by Martínez-Ispizua [58] in eleven lettuce varieties, including landraces and commercial varieties, from 34.26 to 87.01 mg/100 g FW. These differences may be due to both environmental and genetic factors. The growth conditions, including nutrition in the soil, hydroponic growing media, and environmental stresses, have been reported as factors that impact vitamin C biosynthesis in microgreens [74], and may explain the differences found in this study compared to others.

The developmental stage has shown another key factor in vitamin C content depending on the species. Yadav et al. [15] measured the vitamin C content in 9 microgreens and found that while some species (jute and cucumber) had higher vitamin C compared to their mature stages, other species showed similar or lower values (water spinach, *Amaranthus*, bottle gourd, palak, pumpkin, poi, and radish). According to Xiao et al. [14] and Martínez-Ispizua [58], our results showed higher TAA values at the microgreen and baby stages in comparison with their adult counterparts, except for chard. In this species, mature plants showed higher vitamin C compared to the microgreen stage in both local cultivars. Our results confirm that, in general, young plants of these species are a good resource of vitamin C or ascorbic acid. Besides, as microgreens and babies are usually consumed fresh, vitamin C can be largely retained without cooking.

### 3.5. Total Polyphenols

**Figure 6:**
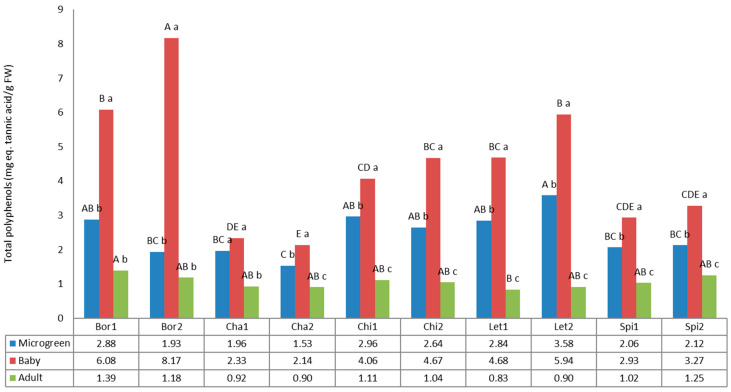
Polyphenols content in the 10 landraces of 5 leafy vegetable crops (borage “Bor”, chard “Cha”, chicory “Chi”, lettuce “Let”, and spinach “Spi”) evaluated at 3 developmental stages (microgreen, baby, and adult). Values are the mean of three replicates, subjected to a one-way ANOVA. Different capital and lowercase letters indicate significant differences between landraces for each developmental stage and between developmental stages for each landrace, respectively, at *p* < 0.05 by Tukey B test. FW: Fresh weight.

Phenolic compounds are one of the most important groups of compounds responsible for various plant bioactivities. Figure 6 shows total polyphenols contents, expressed as mg of tannic acid equivalent (eq.) (TAE). The values for polyphenols statistically differed according to the variety and the developmental stage of the analyzed plants.

Total polyphenols content varied from 8.17 mg TAE/g FW (Bor2, baby) to 0.83 mg TAE/g FW (Let1, adult). All crops showed the highest values at the baby developmental stage, highlighting borage, lettuce, and chicory, followed by the microgreen and adult stages. Especially noteworthy is the high content found in Bor2 at the baby stage, which differed statistically from the rest of the evaluated samples.

Microgreens polyphenols content ranged from 1.53 mg TAE/g FW (Cha2) to 3.58 mg TAE/g FW (Let2), while baby values varied from 2.14 mg TAE/g FW (Cha2) to 8.17 mg TAE/g FW (Bor2). According to Ghoora et al. [65], leafy vegetable microgreens present 2- to 5-fold more nutrients than mature leaves from adult vegetables. This was also supported by Martínez-Ispizua et al. [58], with the phenolic content almost 5-fold higher in seedlings than in adult lettuces regardless of the variability observed among varieties. In our study, this tendency was also proven for the baby stage, with values 2- to 6-fold higher than their mature counterparts; while for microgreens, the values are only 2–3-fold higher than mature plants.

Although phenolic composition has been reported to be highly influenced by cultivar in chicory [75], our results showed a similar phenolic content between the two chicory cultivars. In addition, the other four species did not show differences between the two cultivars either. The results obtained in chicory for adult plants (1.11 and 1.04 mg TAE/g FW), are similar to the state for wild chicory (1.02 mg GAE/g FW), and much higher values were reported for cultivated chicory (0.30 mg GAE/g FW) [54]. The fact that our values for chicory landraces are closer to those of the wild species may be explained because they have not been subjected to formal breeding programs.

Microgreens polyphenols content varied from 1.53 mg TAE/g FW (Cha2) to 3.58 mg TAE/g FW (Let2); higher values have been reported in a study of local microgreens from 3.30 mg GAE/g FW (amaranth) to 22.9 mg GAE/g FW (kohlrabi) [74].

Phenolic compounds for lettuce previously reported by Martínez-Ispizua et al. [58] at the microgreen, baby, and adult stages with mean values of 18.69, 18.43 and 3.91 mg/g DW agree with our results, since we also found the lower values at the adult stage. Nevertheless, we found significant higher values at the baby stage in comparison with the microgreens.

The phenolic compounds have antioxidant properties and are beneficial to human health. Its strong antioxidant efficacy comes from its capacity to scavenge free radicals and indirectly reduce the buildup of reactive oxygen species (ROS) [72]. Phenolic compounds also seem to influence the sensory qualities of microgreens, as it has been correlated with the overall eating quality and several sensory qualities [76]. Our results identify borage, lettuce, and chicory, specifically at the baby stage, as the best sources of these compounds.

### 3.6. Antioxidant Activity

**Figure 7:**
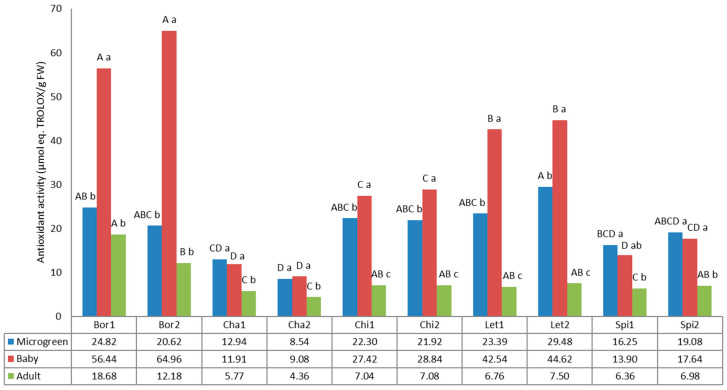
Antioxidant activity in the 10 landraces of 5 leafy vegetable crops (borage “Bor”, chard “Cha”, chicory “Chi”, lettuce “Let”, and spinach “Spi”) evaluated at 3 developmental stages (microgreen, baby and adult). Values are the mean of three replicates, subjected to a one-way ANOVA. Different capital and lowercase letters indicate significant differences between landraces for each developmental stage and between developmental stages for each landrace, respectively, at *p* < 0.05 by Tukey B test. FW: Fresh weight.

Figure 7 shows the antioxidant activity, expressed as µmol eq. Trolox/g FW. The values for antioxidant activity differed statistically according to the variety and the developmental stage of the analyzed plants.

The values varied from 4.36 µmol eq. Trolox/g FW (Cha2, adult) to 64.96 µmol eq. Trolox/g FW (Bor2, baby). Borage, chicory, and lettuce showed the highest values at the baby developmental stage, followed by the microgreen and adult stages, while chard and spinach showed similar values for microgreen and baby, and lower values for the adult developmental stage. The highest antioxidant activity was found for both borage landraces at the baby stage, with values that statistically differed from the rest of the evaluated samples and were more than double the values for the microgreen and adult borage samples. The antioxidant activity followed the same pattern as the phenolic compounds, suggesting that phenols are determinants of the generally increased antioxidant activity. In fact, the cultivars that stood out for polyphenols tended to have a much higher antioxidant activity. This tendency has been previously reported for lettuce [58].

The high antioxidant activity is thought to be one the potential dietary advantages provided by microgreens [71], while some authors have found that mature plants had significantly higher antioxidant activity compared to their microgreens [15]. Our results did not follow this tendency, as the microgreens and baby greens always showed higher values than their adult counterparts; only borage did not show significant differences between microgreens and adult plants.

As previously mentioned for polyphenols, both borage landraces stood out in the baby stage, highlighting their antioxidant potentiality.

## 4. Conclusions

This paper presents a complete study on the nutritional composition of leafy vegetables obtained from local genetic resources, called landraces, at three development stages (microgreen, baby, and adult). The results proved that microgreens and baby greens, a relatively new plant-based functional food, have been shown to be a good source of phytochemicals, better than their adult counterparts. In general, mature plants showed significant lower levels of fatty acids, carotenoids, tocopherols, vitamin C, polyphenols, and antioxidant activities.

The study demonstrated that the date of harvest is important to enhance the vegetable’s nutritional components. We can conclude that, in general, baby greens were a good source of total fatty acids, total polyphenols, α-tocopherol, γ-tocopherol, antioxidant activity and vitamin C (chard and spinach). On the other hand, microgreens showed, in general, the highest values for carotenoids and vitamin C (chicory and lettuce). Both microgreens and baby greens are advantageous because their growth period is much shorter, and their maintenance is considerably lower compared to matured green plants.

The micro-vegetables industry could benefit from these results to promote their qualities, since we have shown that due to their composition they could be consumed for better health. In addition, the species analysed for the first time in this study could suggest expanding the species used for these purposes through the use of local genetic resources. In fact, the study revealed different nutraceutical profiles depending on the crop, although both landraces from each crop behaved in a similar way. Some biochemicals were species-specific: for example, regarding fatty acids, roughanic acid has only been detected in chard and spinach, while stearidonic acid has only been detected in borage, which was also highlighted for its gamma linolenic acid content. These findings highlight the importance of defining genotype-specific nutritional profiles to optimize and exploit the potential of leafy vegetables at different development stages in the micro-vegetables industry.

In conclusion, the results demonstrate the potential of these leafy vegetables as a source of functional compounds and their variability depending on the crop and the developmental stage, reinforcing the requirement of having a varied diet, since each leafy vegetable crop stage offers a unique nutritional profile.

## Figures and Tables

**Table 1:** Plant material used in this study was provided by the Spanish Vegetable Genebank of the Agrifood Research and Technology Centre of Aragón (BGHZ-CITA).

Acronym	Species	Crop	Genebank Code	Plant Description (Differential Traits)
Bor1	*Borago officinalis* L.	Borage	BGHZ3644	Blue flowers
Bor2	*Borago officinalis* L.	Borage	BGHZ5705	White flowers
Cha1	*Beta vulgaris* L.	Chard	BGHZ3760	Green and red leaves with red leaf veins and petioles
Cha2	*Beta vulgaris* L.	Chard	BGHZ7028	Green leaves
Chi1	*Cichorium intybus* L.	Chicory	BGHZ6528	Green leaves
Chi2	*Cichorium intybus* L.	Chicory	BGHZ6529	Green leaves and basal anthocyanin pigmentation
Let1	*Lactuca sativa* L.	Lettuce	BGHZ0366	Green leaves
Let2	*Lactuca sativa* L.	Lettuce	BGHZ7486	Dark green and red leaves
Spi1	*Spinacia oleracea* L.	Spinach	BGHZ6823	Spineless seeds
Spi2	*Spinacia oleracea* L.	Spinach	BGHZ0540	Spiny seeds

## Data Availability

The datasets generated for this study are available on request to the corresponding author.

## Supplementary Materials

The following supporting information can be downloaded at: https://www.mdpi.com/article/10.3390/foods12173173/s1, Table S1: Summary of plant species utilized, developmental stages studied, and quantified compounds. Table S2: Fatty acids [myristic acid (MA, C14:0), palmitic acid (PA, C16:0), palmitoleic acid (PLA, C16:1 9c), roughanic acid (RA, C16:3), margaric acid (MA, C17:0), stearic acid (STA, C18:0), oleic acid (OA, C18:1 9c), cis-vaccenic acid (CV, C18:1 11c), linoleic acid (LA, C18:2 n-6), alfa-linolenic acid (ALA, C18:3 n-3), gamma-linolenic acid (GLA, C18:3 n-6) stearidonic acid (SDA, C18:4, n-3)] identified in the 10 landraces of 5 leafy vegetable crops (borage “Bor”, chard “Cha”, chicory “Chi”, lettuce “Let”, and spinach “Spi”) evaluated at 3 developmental stages (microgreen, baby, and adult). Values are the mean of three replicates per landrace expressed in percentage of total fatty acids. Values for total fatty acid methyl esters (mg FAME/g FW), monounsaturated fatty acids (MUFA), and polyunsaturated fatty acids (PUFA), are also provided; Table S3: Carotenoids (neoxanthin, violaxanthin, zeaxanthin, lutein, 13-Z-β-carotene, 9-Z-β-carotene, all-E-β-carotene, β-carotene total) identified in the 10 landraces of 5 leafy vegetable crops (borage “Bor”, chard “Cha”, chicory “Chi”, lettuce “Let”, and spinach “Spi”) evaluated at 3 developmental stages (microgreen, baby, and adult). Values are the mean of three replicates per landrace.
