# Supplementary material for: Nutraceutical Potential of Leafy Vegetables Landraces at Microgreen, Baby, and Adult Stages of Development
Source: Foods. 2023 Aug 23;12(17):3173. doi: 10.3390/foods12173173 (PMC10486669; doi:10.3390/foods12173173)
Supplement: Supplementary file 1 [file foods-12-03173-s001.zip › foods-2554860-supplementary.pdf]

Table S1. Summary of plant species utilized, developmental stages studied, and quantified compounds.

**Plant species**	Borage	*Borago officinalis* L.
	Chard	*Beta vulgaris* L.
	Chicory	*Cichorium intybus* L.
	Lettuce	*Lactuca sativa* L.
	Spinach	*Spinacia oleracea* L.
**Developmental stages**	Microgreen	Cotyledons and first true leaf. 25-30 days
	Baby	Two-four true leaves. 50-60 days.
	Adult	Commercial stage. 85-95 days.
**Compounds**	Fatty acids	Myristic acid (MA, C14:0), palmitic acid (PA, C16:0), palmitoleic acid (PLA, C16:1 9c), roughanic acid (RA, C16:3), margaric acid (C17:0), stearic acid (STA, C18:0), oleic acid (OA, C18:1 9c), cis-vaccenic (C18:1 11c), linoleic acid (LA, C18:2 n-6), alfa-linolenic acid (ALA, C18:3 n-3), gamma-linolenic acid (GLA, C18:3 n-6), and stearidonic acid (SDA, C18:4, n-3)
	Carotenoids	Neoxanthin, violaxanthin, zeaxanthin, lutein, 13-Z-β-carotene, 9-Z-β-carotene, all-E-β-carotene and β-carotene total
	Tocopherols	α-tocopherol, γ-tocopherol, and δ-tocopherol
	Vitamin C	Expressed as total ascorbic acid (TAA)
	Polyphenols	Expressed as mg eq. tannic acid (TAE)
	Antioxidant activity	Expressed as μmol eq. TROLOX

Table S2. Fatty acids [myristic acid (MA, C14:0), palmitic acid (PA, C16:0), palmitoleic acid (PLA, C16:1 9c), roughanic acid (RA, C16:3), margaric acid (MA, C17:0), stearic acid (STA, C18:0), oleic acid (OA, C18:1 9c), cis-vaccenic (CV, C18:1 11c), linoleic acid (LA, C18:2 n-6), alfa-linolenic acid (ALA, C18:3 n-3), gamma-linolenic acid (GLA, C18:3 n-6) stearidonic acid (SDA, C18:4, n-3)] identified in the 10 landraces of 5 leafy vegetable crops (borage "Bor", chart "Cha", chicory "Chi", lettuce "Let", and spinach "Spi") evaluated at 3 developmental stages (microgreen, baby, and adult). Values are the mean of three replicates per landrace expressed in percentage of total fatty acids. Values for total fatty acid methyl esters (mg FAME / g FW), monounsaturated fatty acids (MUFA), and polyunsaturated fatty acids (PUFA), are also provided.

Acromym	Development stages	MA C14:0	PA C16:0	PLA C16:1 9c	MA C17:0	STA C18:0	RA C16:3	OA C18:1 9c	CV C18:1 11c	LA C18:2 n-6	GLA C18:3 n-6	ALA C18:3 n-3	SDA C18:4 n-3	mg FAME / g FW	MUFA	PUFA
Bor1	Microgreen	0.26	19.75	0.16	0.14	7.05	ND	4.19	0.46	16.15	12.26	26.64	12.47	4.43	4.81	67.53
	Baby	0.31	23.05	0.15	0.08	6.70	ND	2.65	0.41	14.92	9.77	27.31	14.16	5.23	3.21	66.17
	Adult	0.22	19.68	0.26	0.05	6.35	ND	4.36	0.48	14.97	8.31	30.77	14.01	4.27	5.10	68.06
Bor2	Microgreen	0.33	21.66	0.25	0.13	6.48	ND	5.96	0.58	16.50	11.11	26.32	10.29	4.60	6.79	64.22
	Baby	0.51	23.29	0.29	0.13	6.26	ND	4.54	0.51	14.71	8.96	27.67	12.69	5.36	5.33	64.03
	Adult	0.22	21.72	0.27	0.06	6.28	ND	4.28	0.49	14.56	8.17	30.61	12.76	3.86	5.04	66.10
Cha1	Microgreen	0.18	19.40	0.09	0.07	3.15	2.00	1.89	0.37	29.24	0.01	42.85	ND	3.49	2.35	74.10
	Baby	0.28	23.86	0.13	0.08	4.43	1.50	3.30	0.39	23.84	0.04	41.66	ND	5.01	3.82	67.05
	Adult	0.23	21.55	0.25	0.05	4.22	1.98	4.08	0.45	21.86	0.03	44.85	ND	3.72	4.78	68.72
Cha2	Microgreen	0.32	20.83	0.09	0.10	5.77	1.59	2.34	0.32	24.96	0.02	43.05	ND	4.24	2.75	69.62
	Baby	0.26	23.20	0.14	0.09	4.72	1.55	3.26	0.36	24.45	0.04	41.46	ND	4.59	3.76	67.50
	Adult	0.22	21.63	0.16	0.05	4.58	1.85	3.65	0.38	21.98	0.01	45.02	ND	3.42	4.18	68.86
Chi1	Microgreen	0.27	16.78	0.13	0.10	4.51	ND	1.83	0.20	20.11	0.08	55.33	ND	6.20	2.16	75.52
	Baby	0.40	22.14	0.24	0.07	3.76	ND	1.52	0.23	18.71	0.18	52.22	ND	8.53	1.98	71.11
	Adult	0.22	17.71	0.26	0.05	3.49	ND	2.01	0.29	18.29	0.18	56.94	ND	5.31	2.56	75.41
Chi2	Microgreen	0.23	17.09	0.14	0.10	4.55	ND	1.57	0.22	20.72	0.08	54.61	ND	5.78	1.92	75.41
	Baby	0.50	22.41	0.21	0.08	3.94	ND	1.57	0.21	20.87	0.16	49.53	ND	7.51	1.99	70.56
	Adult	0.26	18.20	0.33	0.05	4.21	ND	2.62	0.35	18.45	0.14	54.84	ND	4.78	3.30	73.42

Acromym	Development stages	MA C14:0	PA C16:0	PLA C16:1 9c	MA C17:0	STA C18:0	RA C16:3	OA C18:1 9c	CV C18:1 11c	LA C18:2 n-6	GLA C18:3 n-6	ALA C18:3 n-3	SDA C18:4 n-3	mg FAME / g FW	MUFA	PUFA
Let1	Microgreen	0.36	17.26	0.18	0.08	4.53	ND	1.32	0.21	23.50	0.04	50.35	ND	5.63	1.71	73.89
	Baby	0.66	22.53	0.24	0.07	5.60	ND	1.17	0.23	23.81	0.03	45.15	ND	7.76	1.64	69.00
	Adult	0.28	17.04	0.29	0.04	4.09	ND	1.89	0.29	18.45	0.01	57.02	ND	4.71	2.47	75.48
Let2	Microgreen	0.37	16.67	0.21	0.09	3.95	ND	1.61	0.30	21.98	0.05	53.34	ND	5.78	2.12	75.36
	Baby	0.59	20.75	0.35	0.09	5.02	ND	2.03	0.31	22.30	0.04	47.99	ND	7.21	2.70	70.33
	Adult	0.26	16.70	0.40	0.05	3.59	ND	2.15	0.42	18.90	0.01	56.93	ND	4.51	2.96	75.84
Spi1	Microgreen	0.26	17.67	0.22	0.11	4.26	5.38	4.78	0.94	17.41	0.03	48.14	ND	4.71	5.94	70.96
	Baby	0.33	19.68	0.24	0.09	4.33	5.47	4.26	1.02	15.68	0.03	48.23	ND	6.03	5.52	69.42
	Adult	0.25	16.86	0.24	0.04	3.51	7.33	4.22	1.03	11.12	0.02	54.77	ND	5.91	5.49	73.24
Spi2	Microgreen	0.23	16.22	0.19	0.10	3.34	6.68	5.20	1.19	16.24	0.01	49.99	ND	5.23	6.58	72.92
	Baby	0.40	20.87	0.26	0.12	4.99	5.12	4.47	1.36	14.92	0.03	46.88	ND	6.43	6.09	66.95
	Adult	0.25	16.87	0.24	0.03	3.39	7.23	4.47	1.19	10.37	0.01	55.40	ND	6.17	5.89	73.01

ND: Not Detected

Table S3. Carotenoids (neoxanthin, violaxanthin, zeaxanthin, lutein, 13-Z-β-carotene, 9-Z-β-carotene, all-E-β-carotene, β-carotene total) identified in the 10 landraces of 5 leafy vegetable crops (borage "Bor", chart "Cha", chicory "Chi", lettuce "Let", and spinach "Spi") evaluated at 3 developmental stages (microgreen, baby, and adult). Values are the mean of three replicates per landrace.

Acromym	Development stages	Neoxanthin µg/g FM	Violaxanthin µg/g FM	Zeaxanthin µg/g FM	Lutein µg/g FM	13-Z-β-carotene µg/g FM	9-Z-β-carotene µg/g FM	All-E-β-carotene µg/g FM	Total β-carotene µg/g FM	Total carotenoids µg/g FM
Bor1	Microgreen	10.69	36.86	4.40	38.26	2.56	5.21	35.09	42.85	133.06
	Baby	8.21	16.28	1.34	30.34	2.26	4.25	27.45	33.95	90.12
	Adult	10.70	26.72	0.74	35.23	3.09	5.92	43.86	52.88	126.28
Bor2	Microgreen	10.42	35.59	3.16	37.53	2.31	4.69	30.78	37.78	124.49
	Baby	7.71	15.12	1.34	29.18	2.16	4.27	27.32	33.74	87.08
	Adult	10.60	28.30	0.55	34.02	3.14	5.81	37.79	46.74	120.20
Cha1	Microgreen	6.16	23.62	2.06	34.64	2.26	4.19	24.25	30.70	97.19
	Baby	5.13	11.09	2.38	27.16	2.00	4.26	23.56	29.82	75.57
	Adult	5.43	13.70	1.36	24.66	2.30	4.91	26.53	33.73	78.88
Cha2	Microgreen	8.68	29.96	1.67	38.33	2.12	4.22	27.61	33.94	112.59
	Baby	6.96	12.54	1.68	29.20	2.13	3.90	24.36	30.39	80.78
	Adult	4.43	11.08	1.35	22.12	2.14	4.42	23.31	29.87	68.85
Chi1	Microgreen	11.48	50.63	3.65	56.82	3.82	8.62	47.04	59.48	182.07
	Baby	10.84	20.71	2.06	45.20	3.62	6.62	40.95	51.19	129.99
	Adult	11.62	26.70	1.29	41.34	3.52	7.27	40.82	51.62	132.57
Chi2	Microgreen	11.05	48.66	4.25	58.97	3.59	7.79	43.66	55.04	177.97
	Baby	10.40	13.61	2.67	43.10	6.27	5.40	34.84	46.50	116.30
	Adult	12.78	19.56	0.93	43.27	6.78	7.16	35.62	49.55	126.10
Let1	Microgreen	10.39	47.59	7.33	29.86	2.88	6.19	36.30	45.38	140.54
	Baby	7.97	11.81	5.74	19.21	5.25	4.52	23.09	32.87	77.60
	Adult	14.69	31.10	2.09	26.43	4.03	6.48	42.17	52.68	126.99

Acromym	Development stages	Neoxanthin µg/g FM	Violaxanthin µg/g FM	Zeaxanthin µg/g FM	Lutein µg/g FM	13-Z-β-carotene µg/g FM	9-Z-β-carotene µg/g FM	All-E-β-carotene µg/g FM	Total β-carotene µg/g FM	Total carotenoids µg/g FM
Let2	Microgreen	12.54	55.42	6.12	33.19	3.47	7.23	43.99	54.69	161.95
	Baby	8.48	12.27	4.16	20.49	4.13	5.74	29.96	39.84	85.24
	Adult	11.20	28.68	2.45	23.65	3.41	7.08	39.55	50.04	116.02
Spi1	Microgreen	13.86	44.18	4.56	47.39	2.94	5.49	40.92	49.35	159.34
	Baby	11.18	27.55	3.87	41.39	3.04	5.68	37.27	46.00	129.98
	Adult	18.74	41.47	8.73	70.39	6.35	12.59	80.53	99.46	238.79
Spi2	Microgreen	15.09	47.35	4.14	52.54	3.79	7.17	49.37	60.33	179.45
	Baby	7.97	18.42	3.99	36.53	2.71	5.79	34.20	42.71	109.63
	Adult	11.71	34.60	7.40	51.07	4.68	10.88	59.20	74.76	179.53
